# Perceived learning curve of the nursing team using the da vinci XI surgical system in a Brazilian university hospital

**DOI:** 10.1007/s11701-026-03765-z

**Published:** 2026-07-31

**Authors:** Ricardo de Oliveira Meneses, Letícia Perfeito Ramos, Lisandra Rodrigues Risi, Rosilene Alves Ferreira, Camila Castanho Cardinelli, Fernanda Ferreira e Silva

**Affiliations:** Rio de Janeiro State University, Rio de Janeiro, Brazil

**Keywords:** Learning curve, Perioperative Nursing, Robotic Surgery, Patient Safety, Education, Nursing, Continuing, da Vinci Xi

## Abstract

This qualitative exploratory study evaluated 25 nursing team members experienced in Robotic-Assisted Surgery (RAS) at a Brazilian university hospital. Data collection used a validated instrument (CVI = 1.0), and Bardin’s thematic analysis identified three competency domains: clinical practice/system configuration, management of inputs/logistics, and handling of complications/learning curve indicators. Participants were predominantly female (88%), averaging three years of RAS experience with high heterogeneity (CV ≈ 68%). All participants performed patient positioning and endoscope connection; 92% managed electrosurgical settings; and 80% handled decoupling and pre-cleaning. Basic system proficiency required a weighted average of 16 procedures, whereas safe intraoperative performance required 13.5 weeks of immersion. Emergency undocking was reported by 28% of participants, primarily caused by power failures or system instability. The perceived learning curve was non-linear and dependent on procedure frequency, specialty complexity, and institutional support. In conclusion, the learning curve is multidimensional and cannot be reduced to single targets. Brazilian nursing teams operate beyond formal regulatory boundaries, highlighting the need for competency-based training, specialty exposure, and emergency simulation protocols. The gap between perceived proficiency and objective thresholds shows self-reported learning reflects operational familiarity rather than complete clinical safety. Standardizing emergency undocking preparedness remains an essential patient safety priority. These findings establish a baseline for standardized nursing RAS training programs.

## Introduction

The introduction of robotic-assisted surgery (RAS) has revolutionized modern surgical practice, offering well-documented benefits such as reduced blood loss, faster patient recovery, and superior visualization for the surgeon [1–2]. However, the integration of these sophisticated platforms into the operating room (OR) has fundamentally altered the perioperative environment, shifting traditional workflows and imposing significant technical demands on the surgical team [2]. While much of the early literature focused on the surgeon’s transition to robotics, there is an increasing recognition that perioperative nurses are central players in coordinating these complex procedures, yet they face a distinct and challenging learning curve [2–3].

The adaptation process for nurses in RAS is characterized by the need to transition from traditional surgical assistance to a role requiring high technological competence [2, 4]. Nurses must master specific tasks, including the complex configuration and preparation of the robotic system, precise patient positioning, which is critical to avoid peripheral nerve injuries, and troubleshooting equipment malfunctions under pressure. Furthermore, the physical separation of the surgeon at the console from the bedside team necessitates a shift in communication dynamics, requiring nurses to adopt augmented, task-specific verbal strategies to maintain situational awareness and ensure patient safety [1, 3, 5].

Despite the complexity of these new responsibilities, evidence suggests a significant gap in formal nursing education for robotics [1–3]. Many nurses currently acquire these high-stakes skills through informal “master-apprentice” relationships or self-directed information seeking, leading to feelings of uncertainty, stress, and anxiety regarding potential harm to the patient. Studies examining the adaptation timeline indicate that while many nurses feel “adapted” within three months, others require six months or more to achieve full proficiency, particularly in the absence of structured professional development programs [3–6].

Factors such as individual innovativeness, the degree to which a professional is open to new ideas and technologies, have been shown to significantly correlate with a shorter adaptation period [2]. However, the lack of standardized curricula remains a primary barrier to optimizing the nursing learning curve [5–6]. Understanding the nuances of this curve is essential for developing evidence-based training protocols that reduce surgical time, lower costs, and, most importantly, maximize the safety and quality of care in the robotic operating room.

Given this context, the need for structured training and studies that expand knowledge about the nursing learning curve in RAS is highlighted, as this field is still being consolidated in the national context [7–9]. The learning curve, as a basis for technical improvement, underpins the research question of this study: How do nursing team in a university hospital perceive their learning curve for the da Vinci Xi Surgical system [10]? .

## Method

### Study design and settings

The investigator (L.P.R., female, nurse, resident nurse), experienced in qualitative clinical research, conducted all interviews. Prior to the study, no relationship existed between researcher and participants. Participants were informed about the researcher’s professional background and the academic goals of the study.

This exploratory descriptive study with qualitative and quantitative components was conducted using the Consolidated Criteria for Reporting Qualitative Research (COREQ) [11]. The study was carried out in the surgical center of a university hospital in Brazil, recognized as a teaching, research and care institution. The hospital provides general care to populations requiring health services and represents a large teaching-care complex relevant to national training in health professions.

In this study, learning curve refers to the nursing team’ (NT) perceived exposure requirements and self-reported operational competency development. Objective performance indicators, such as operative time by case sequence, were not measured.

### Participants

**Fig. 1:**
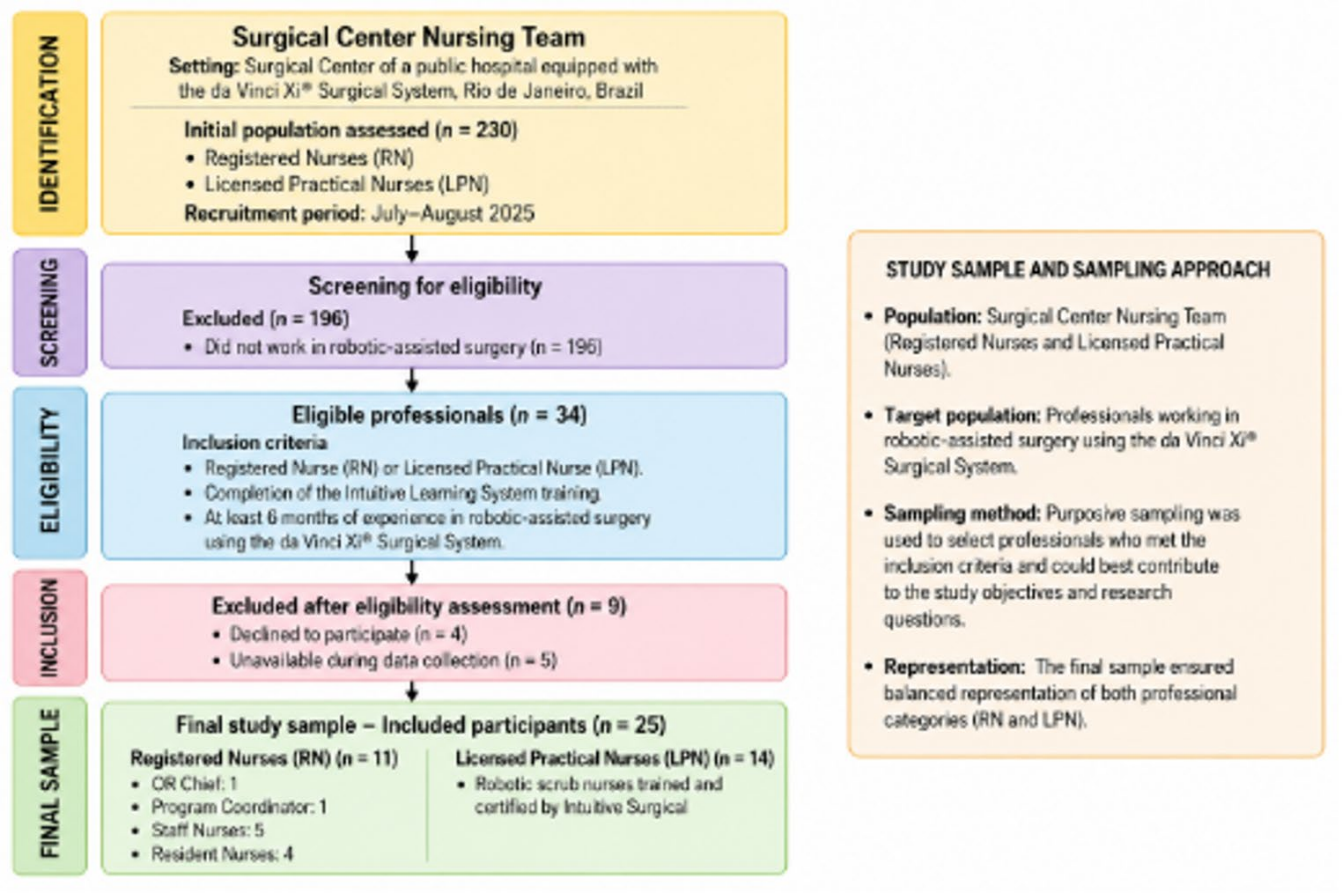
Participants selection flow. Rio de Janeiro, Brazil, 2025

The study population comprised the robotic NT previously trained through the Intuitive Learning System for the da Vinci Xi platform. The participant selection process is illustrated in Fig. 1. The final group included 25 professionals, consisting of 14 Licensed Practical Nurse (LPN) and 11 registered nurses (RN). The inclusion criteria were completion of training through the Intuitive Learning System and at least six months of experience in RAS in a hospital surgical center using the da Vinci Xi Surgical System.

### Data collection

Data were collected from July to August 2025 through expert-validated semi-structured interviews. All face-to-face interviews took place in a quiet, private room at the university hospital. No third parties were present. No repeat interviews were conducted. All sessions were audio-recorded, lasting an average of 20 min, and the researcher took detailed field notes immediately afterward.

The data collection instrument was evaluated by a panel of five expert judges, independent from the study sample. The inclusion criteria for the expert judges required: specialists with active experience in RAS, to be specialists in both the Surgical Center, with a specific focus on robotics. We also included nurses who completed the robotic training program but were not active in clinical practice, provided they knew how to manipulate the system. Lastly, Consent Form was not required for these participating experts. The judges assessed item clarity, relevance, and pertinence based on a review study and the Intuitive Learning manual. Content validity was verified using the Content Validity Index (CVI) with values ≥ 0.80 considered acceptable.

The instrument included three sections: sociodemographic data, nursing duties and competencies in robotic procedures, and perceptions of the learning curve. It also investigated previous experience with emergency undocking and the minimum number of procedures considered necessary to understand robotic system operation.

### Data analysis and reporting

Quantitative variables, such as number of procedures and experience time, were analyzed using absolute and relative frequencies and weighted averages. Two independent researchers (R.O.M. and L.P.R.) coded the transcripts. It was analysed using Bardin’s content analysis [12]. The analysis used constant comparison between RN and LPN, with thematic saturation achieved when no new relevant codes emerged. MAXQDA software supported coding and frequency analysis.

Representative, anonymized participant quotes (e.g., “RN 1”) are presented in the results section to support the findings. Discrepancies in coding and divergent, minor cases were discussed and resolved by consensus to ensure analytical consistency. The final coding framework was organized into categories related to technical activities, emergency response and perceived competency development.

### Ethical considerations

This study was performed in accordance with the ethical principles of the Declaration of Helsinki. The study was approved on December 12th, 2024 by Ethics Committee of Rio de Janeiro State University (Opinion No. 7.291.321). Participants confirmed their acceptance by reading and signing the consent form.

**Fig. 2:**
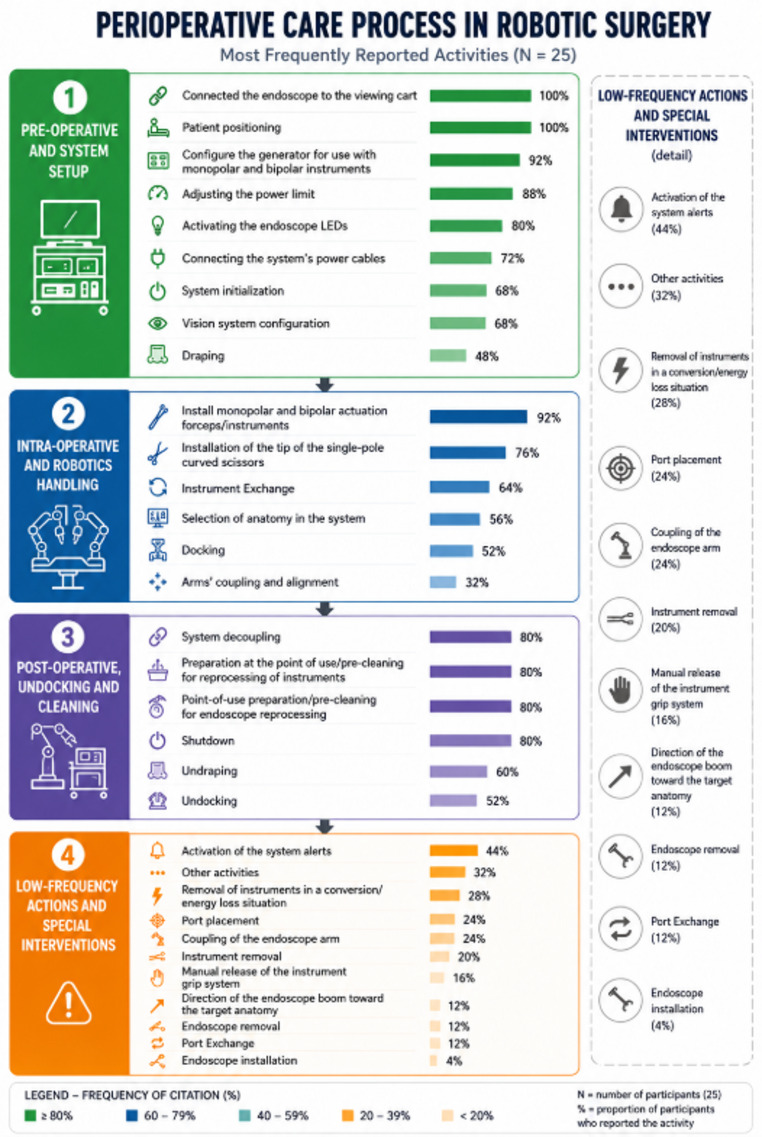
Expression of duties performed by the nursing team in the operating room using the da Vinci Xi surgical system, as recognized in the surgical setting (*n* = 25). Rio de Janeiro, Brazil, 2025. *This item allows multiple selections

An Artificial Intelligence tool was used just to optimize the layout and formatting of Fig. 2 to improve readability for the reader. The tool did not generate, analyze, or interpret any scientific data. All table content remains original and was fully verified by the authors.

### Results

All instrument items showed full agreement among the judges, with a CVI of 1.0 indicating excellent content validity. The analysis was performed manually, with individual evaluation records and subsequent consensus among experts.

Twenty-five interviews were conducted with the NT working in the RAS operating room. Among the participants, 22 were women and three were men, with ages ranging from 20 to 60 years. The NT profile and experience in RAS are described in Table 1. It presents participants’ educational level, healthcare experience, experience in RAS, function in the robotic operating room and participation in RS procedures. It also summarizes the heterogeneity of the NT including professionals at different stages of the RS learning curve.

**Table 1:** Nursing team profile and experience in robotic-assisted surgery (*n* = 25). Rio de Janeiro, Brazil, 2025

VARIABLE	*n*	%
**Educational level**		
Licensed practical nurses with higher education and postgraduate studies	11	44
Licensed practical nurses with surgical instrumentation course	4	16
Higher education with postgraduate studies	4	16
Technical and higher education	3	12
Higher education Only	2	8
Nursing technician Only	1	4
**Healthcare experience**		
< 10 years	10	40
10 to 19 years	9	36
20 to 30 years	5	20
> 30 years	1	4
Experience in RS		
One year or less	7	28
2 years	4	16
3 years	5	20
4 years	1	4
5 years	4	16
6 years	4	16
Function in the robotic operating room		
Circulating and scrub professionals	14	56
Robotic operating room nurses	11	44
**Participation in robotic surgical procedures**		
25 to 50 procedures	10	40
50 to 75 procedures	3	12
75 to 100 procedures	3	12
100 to 150 procedures	3	12
> 150 procedures	6	24

RAS experience showed a mean of approximately 3 years, median of 3 years, standard deviation of approximately 2.05, and coefficient of variation of approximately 68%, indicating heterogeneity in the NT’s experience.

The qualitative data were divided into three categories: Clinical practice and robotic system configuration, Management of Inputs, Infrastructure, and Logistics Processes, and Management of Complications and Learning Curve Indicators.

### Clinical practice and robotic system configuration

The first category addresses the practical operation of the robotic system and the peripheral equipment required for surgery.

All participants (100%) assisted with patient positioning and connected the endoscope to the vision cart. Additionally, 92% (*n* = 23) managed electrosurgical tasks, specifically configuring generators and installing monopolar and bipolar forceps. Adjusting energy limits was also highly prevalent, reported by 88% (*n* = 22) of the sample.

Furthermore, 80% (*n* = 20) performed endoscope LED activation, system decoupling, shutdown procedures, and point-of-use pre-cleaning of instruments. Ultimately, over half of the nursing technicians associated these robotic operations with their general professional knowledge (Fig. 2).

### Management of inputs, infrastructure, and logistics processes

This category includes the administrative, organizational, and oversight functions required to ensure surgical safety and feasibility.

Additionally, 32% of the participants reported other duties, such as recording intraoperative images and configuring pneumatic parameters. These tasks involved activating the insufflator, adjusting pneumoperitoneum pressure, and arranging the operating room layout.

Regarding logistical support, these professionals managed essential surgical supplies, monitored instrument lifespan, and provided specialized orthoses, prostheses, and materials. They also assisted surgeons in utilizing new instruments during the procedure.

Finally, administrative and technical support included configuring console ergonomics and registering surgeon data in the Strattner cloud. These activities also encompassed restoring the sterile barrier if compromised, managing damaged materials, and notifying the manufacturer for analysis.

#### N6

*“That’s very specific*,* but*,* for example*,* assisting a surgeon in using a specific forceps*,* I think that’s very specific too*,* but it has happened in some situations that I’ve had to teach the surgeon how to activate a stapler*,* use a Vessel Sealer*,* because the surgeon doesn’t necessarily know how to handle them*,* and that ends up falling a little within the scope of the operating room nurse’s role in the functionality of the materials.”*

### Management of Complications and Learning Curve Indicators

This category covers fault response, emergency shutdowns, and the estimated time required to achieve technical proficiency.

### Management of Complications and Conversions

Regarding emergency instrument removal during outages or conversions, seven professionals reported encountering this scenario. Four participants attributed emergency undocking to power failures, system overheating, or unresponsiveness.

#### RN2

*“Yes*,* there was a power failure […] I had to undock the robot. The surgery was converted […] I explained to the surgeon that the system had restarted*,* but there was a risk of instability. For equipment safety*,* he preferred to convert to an open procedure.”*

Additionally, some professionals assisted in conversions from robotic surgery to laparotomy. These events were primarily driven by patient clinical conditions, such as anatomical incompatibility or active intracavitary bleeding.

### Technical proficiency and number of procedures

Participants reported diverse perceptions regarding the minimum number of procedures needed to understand the robotic system. Most indicated that five procedures were sufficient (32%), followed by ten (28%) and twenty (20%).

Smaller proportions reported needing thirty procedures (8%), two (4%), forty (4%), or between 50 and 100 (4%). Based on the weighted averages of these responses, the estimated minimum for basic proficiency was approximately 16 procedures.

#### N2

*“After 20 to 30 procedures*,* you understand it better […] surgical times in robotics are not standardized. There is docking*,* undocking*,* and specific procedure demands. […] This volume provides a sufficient level of knowledge to handle the main issues.”*

### Immersion time and dexterity acquisition

Regarding the immersion time needed to acquire dexterity, 40% of participants indicated either one or three months as sufficient. Furthermore, 28% reported two months, while smaller proportions indicated six months (12%), one intensive week (8%), twelve months (8%), or eight months (4%).

The weighted average of these responses resulted in an estimated immersion time of 13.5 weeks (approximately 3.4 months) for safe performance.

#### LPN4

*“I think a year is enough because of clinical variability. Each specialty changes patient positioning*,* grippers*,* and adjacent materials. To master all clinics*,* it takes time.”*

These findings highlight a relatively short but intense learning phase. Proficiency is predominantly achieved through consistent exposure and high practice frequency.

#### N2

*“To handle this alone*,* a nurse needs 20 to 30 procedures. However*,* to fully master the technology and best practices*,* I believe it takes up to 6 months.”*

## Discussion

This study mapped the perceived learning curve of a nursing team engaged in robot-assisted surgery using the da Vinci Xi Surgical System in a Brazilian university hospital. By characterizing both the technical activities performed and the subjective thresholds reported for proficiency, the findings contribute to a growing but still limited body of evidence on nursing competence in perioperative robotic settings. The results indicate that the learning curve for NT is not a single, objective metric but a multidimensional construct shaped by role scope, exposure frequency, institutional support, and task complexity.

The three competency domains identified reflect a perioperative role that extends well beyond passive support. The finding that all participants were involved in patient positioning and endoscope connection, and that 92% managed electrosurgical configurations, is analytically significant for two reasons. First, it reveals a wider scope of practice than typically documented for nursing team in national guidance, where positioning and electrosurgical management are often described primarily as registered nurse responsibilities [8, 13]. This gap between formal role descriptions and actual clinical practice has been documented in the national context and reinforces the urgency of legally grounded scope-of-practice frameworks, such as the COREN Opinion 003/2024 [14].

Second, the near-universal involvement in patient positioning aligns with international qualitative evidence. Bjøro et al. [15] describe RAS positioning, specially in steep Trendelenburg, as a high-stakes, multidisciplinary activity requiring proactive leadership from the NT. However, those studies focus on RN, not LPN, underscoring the distinctive profile of the Brazilian robotic NT. The centrality of the ELPO scale in risk stratification [16] and the protocol evidence for skin injury prevention in robotic urology [6] reinforce that positioning competence must be formally incorporated into NT training curricula, given its documented complications including pressure injuries, neuropathies, and hemodynamic instability.

The 80% involvement in system decoupling, LED activation, and point-of-use pre-cleaning further indicates that Brazilian NTs perform tasks that in other health systems are divided among multiple roles or are assigned to other categories. Rather than interpreting this as task overload, it can be read as an indicator that the Brazilian robotic NT functions as a de facto multifunctional unit. A model with both efficiency advantages and training cost implications.

The central empirical contribution of this study lies in quantifying the self-perceived learning curve: a weighted average of approximately 16 procedures for basic system understanding and 13.5 weeks (about 3.4 months) of clinical immersion for safe performance. These figures merit careful analytical interpretation.

The 16-procedure threshold partially converges with evidence from surgical outcomes research. Zeuschner et al. [17] demonstrated that the bedside surgical assistant’s experience, measured by case volume, exerts an independent effect on perioperative outcomes in robotic partial nephrectomy, with meaningful improvement observed after approximately 15 cases. Similarly, Yao et al. [18] identified at least eight cases as the threshold for equipment operation mastery in itinerant nurses trained on Ti-robot-assisted spinal surgery, suggesting that isolated task thresholds may be shorter than holistic proficiency thresholds. This is analytically important: the 16-procedure perception in the present study likely reflects a global operational sense of system familiarity rather than task-specific mastery, and may underestimate the number of cases required for full proficiency across all NT responsibilities.

Conversely, data on draping and docking of the da Vinci Xi system showed that dedicated nursing teams reached a performance plateau only after 18 to 21 cases [19], suggesting that setup-specific tasks alone may require more experience than the overall perceived threshold of 16. For more complex outcome indicators, such as reduction of operative time or complication rates, the learning curve is considerably longer. Andersson et al. [20] found that the operative time plateau in benign robotic hysterectomy occurred after 50 procedures, while reduction in complication rates required 150 procedures. This discrepancy between perceived and empirically measured proficiency thresholds suggests a systematic underestimation by participants, likely driven by the operational focus of their daily tasks rather than clinical outcome awareness.

The 3.4-month immersion estimate as a proxy for safe performance is a finding not validated in the broader literature. Available studies on nursing proficiency in robotic surgery report case-count milestones rather than calendar-based targets [17–18, 21]. This discrepancy has theoretical implications: it suggests that NTs conceptualize their training trajectory primarily through time and routine exposure rather than through deliberate procedural counting. A pattern that may be reinforced by the high heterogeneity of experience in the team (coefficient of variation of approximately 68%). When case frequency is irregular, time becomes the most accessible proxy for experience. Training programs should, however, prioritize case-count and competency-based assessment over calendar duration, as the literature consistently shows that volume and calendar time are not interchangeable when case frequency varies [21].

The wide range of self-reported proficiency thresholds, from two procedures to 50–100 procedures, and the heterogeneity of team experience further reinforce that the learning curve in this setting is not linear, homogeneous, or generalizable. Factors including prior experience with laparoscopic procedures, specialty variability, and institutional caseload volume all modulate individual trajectory. This aligns with the non-linear, contextual learning model described by Wu et al. [22] in their CUSUM-based analysis of single-site robotic prostatectomy.

Seven participants (28%) reported having encountered emergency undocking scenarios, primarily due to power failures and system instability. This prevalence is clinically meaningful in a team of 25 professionals and points to a concrete competency demand that the literature has consistently identified as undertrained and under-protocolized.

Almeida et al. [23] demonstrated that structured simulation training in the RULES (Robotic Undocking for Life Emergency Support) protocol significantly improved team communication, coordination, and undocking time in Brazilian robotic surgery centers, directly relevant to the findings of the present study. Ballas et al. [24] similarly showed that a simulation-based emergency undocking curriculum improved critical action completion rates and team performance. Carlos and Saulan [25] emphasized that emergency preparedness in robotic surgery demands systematic planning, protocol adoption, and team rehearsal. The fact that the NTs in this study managed these events largely without formalized protocols represents an institutional safety gap that must be addressed as a priority in training program design.

Surgical conversion to laparotomy, reported by several participants as a scenario they had encountered, adds another dimension to this competency domain. Conversion events are often driven by patient clinical conditions or technical limitations and require rapid, coordinated team response, including instrument removal, sterile field management, and communication with the surgical team. The NT’s competence in these moments depends not only on technical skill but on distributed situational awareness, a non-technical competency that deliberate practice and simulation can develop [26, 27].

Taken together, the findings converge on a consistent implication: the learning curve for NTs in robotic surgery is real, variable, and trainable. But, it requires structured, competency-based programs rather than unguided time-in-service. The identified domains of practice map directly onto the curriculum content established by the Delphi study of Møller et al. [26], which covers system operation, patient positioning, sterile technique, emergency response, and interdisciplinary communication. The systematic review by the same group [27] found that training programs in robotic OR nursing are heterogeneous and often unvalidated, a limitation that characterizes the national context as well.

The perceived learning curve data from this study can serve as a baseline for designing institutional training programs that define minimum case exposure, competency checkpoints, and structured simulation components. Nicolas et al. [28] demonstrated that accumulated procedural experience systematically reduces operative time and improves OR efficiency in robotic bariatrics; similar workflow optimization benefits can be expected when NT training is formalized and caseload is deliberately managed. Incorporating emergency undocking and conversion drills into these programs, supported by evidence-based protocols [23], would address the identified gap in emergency preparedness.

### Study limitations

This study has several limitations that impact its direct application to clinical practice. First, because data were gathered from a single hospital, the specific institutional structure and high-volume workflow may not reflect conditions of other centers. Similarly, evaluating a single robotic platform without differentiating between surgical specialties limits the insights gained. Specialties involve distinct patient positioning, docking configurations, and procedural demands, making specialty-specific learning curves necessary in practice.

Another critical constraint is that skill acquisition was assessed solely through self-reported perceptions rather than objective performance metrics. In a clinical setting, self-perception is prone to bias and cannot replace concrete indicators like operative times, docking speed, complication rates, or validated competency evaluations.

Finally, the small sample size reduces the statistical power of the quantitative findings and prevents meaningful subgroup analyses based on staff experience level. While these findings offer initial context on nursing workflows, future research using objective metrics, multi-center designs, and specialty-stratified data is essential to establish practical guidelines for robotic training programs.

## Conclusion

This study characterized the perceived learning curve of the nursing team (NT) using the da Vinci Xi Surgical System. Results indicate that the learning curve is a multidimensional construct shaped by specific operational conditions. These lessons can be applied to systems similar to the da Vinci Xi.

Mapping NT activities across three competency domains revealed an expanded scope of practice compared to Brazilian regulatory frameworks. The near-universal involvement in patient positioning and electrosurgical management highlights substantial safety-critical responsibilities, underscoring the need for updated national competency standards.

Participants reported learning thresholds of approximately 16 procedures for basic system understanding and 13.5 weeks for safe performance. These subjective thresholds are lower than literature-documented plateaus for objective indicators, such as operative time and complication rates. This disparity reflects distinct measurement referents: subjective scores measure operational familiarity, whereas objective metrics capture clinical outcome stabilization. Both dimensions are complementary and essential for training program design.

High team heterogeneity confirms that the learning curve is non-linear, individually variable, and caseload-dependent. This variation poses structural risks regarding asymmetric performance, which requires competency-based assessments rather than reliance on seniority.

Emergency response in robotic surgery requires defined roles and regular rehearsal. Integrating simulation-based emergency training and validated decoupling protocols into nursing curricula is therefore a critical patient safety requirement.

In conclusion, robotic nursing proficiency cannot be defined just by case volume or tenure. Effective training programs must be competency-based, structured around explicit benchmarks, stratified by task domain and specialty, and inclusive of emergency simulations. These findings establish a practitioner-derived baseline to design and evaluate robotic nursing programs.

Future research should employ longitudinal, multicenter designs tracking objective performance across key milestones. Studies should include registered nurses and technicians to analyze specialty-specific trajectories. Developing and validating standardized competency assessment instruments for robotic surgical nursing remains an immediate priority.

## Data Availability

No datasets were generated or analysed during the current study.

## References

[1] Martins RC, Trevilato DD, Jost MT, Caregnato RCA (2019) Nursing performance in robotic surgeries: integrative review. Rev Bras Enferm 72(3):795–800. 10.1590/0034-7167-2018-0426 31269148 10.1590/0034-7167-2018-0426

[2] Pires SM, Maurício AR, Jerónimo L, Teixeira B, Ramos A, Gomes I, Sá E (2025) Nursing interventions to promote safety in robotic surgery: A systematic literature review. J Perioper Nurs 38(1):e29–e37. 10.26550/2209-1092.1374

[3] Porto CST, Catal E (2021) A comparative study of the opinions, experiences and individual innovativeness characteristics of operating room nurses on robotic surgery. J Adv Nurs 77:4755–4767. 10.1111/jan.15020 34423468 10.1111/jan.15020

[4] Uslu Y, Altınbaş Y, Özercan T, van Giersbergen MY (2019) The process of nurse adaptation to robotic surgery: A qualitative study. Int J Med Robot 15(4):e1996. 10.1002/rcs.1996 30884169 10.1002/rcs.1996

[5] Lee L, Grewwnway K, Schutz S (2024) What do nurses experience in communication when assisting in robotic surgery: an integrative literature review. J Robot Surg 18:50. 10.1007/s11701-024-01830-z 38280076 10.1007/s11701-024-01830-z PMC10822005

[6] Celik SS, Koken ZO, Canda AE et al (2023) Experiences of perioperative nurses with robotic-assisted surgery: a systematic review of qualitative studies. J Robotic Surg 17:785–795. 10.1007/s11701-022-01511-97 10.1007/s11701-022-01511-9 36542241

[7] Harris M, Bannon A, Collins JW (2025) Procedural robotic surgery training: a UK pan-specialty trainee Delphi consensus study. J Robot Surg 19(1):501. 10.1007/s11701-025-02582-0 40835797 10.1007/s11701-025-02582-0 PMC12367954

[8] Pinto EV, Lunardi LS, Treviso P, Botene DZA (2018) Atuação do enfermeiro na cirurgia robótica: desafios e perspectivas. Revista SOBECC 23(1):43–51. 10.5327/Z1414-4425201800010008

[9] Araujo PHXN, Pêgo-Fernandes PM (2023) Robotic surgery training. Sao Paulo Med J 141(5). 10.1590/1516-3180.2022.1415310823 10.1590/1516-3180.2022.1415310823 PMC10619944 37909546

[10] Anzanello MJ, Fogliatto FS (2007) Curvas de aprendizado: estado da arte e perspectivas de pesquisa. Gestão Produção 14(1):109–123. 10.1590/S0104-530X2007000100010

[11] Tong A, Sainsbury P, Craig J (2007) Consolidated criteria for reporting qualitative research (COREQ): a 32-item checklist for interviews and focus groups. Int J Qual Health Care 19(6):349–357. 10.1093/intqhc/mzm042 17872937 10.1093/intqhc/mzm042

[12] Bardi L (2016) Content Analysis. Edições 70, São Paulo

[13] Minasio Júnior CR, Farineli EMM, Tiago LA, Giuntini PB (2025) Papel do enfermeiro em cirurgias robóticas no período perioperatório. Revista Acadêmica Online 11(56):e433. 10.36238/2359-5787.2025.v11n56.433

[14] Conselho Regional de Enfermagem de São Paulo (2024) Parecer COREN-SP Nº 003/2024: Atuação de profissionais de Enfermagem em cirurgia robótica. COREN-SP, São Paulo. https://portal.coren-sp.gov.br/wp-content/uploads/2024/06/PARECER-COREN-SP-No-003-2024.pdf

[15] Bjøro B, Ballestad I, Rustøen T, Fosmark MH, Bentsen SB (2023) Positioning patients for robotic-assisted surgery: A qualitative study of operating room nurses’ experiences. Nurs Open 10(2):469–478. 10.1002/nop2.1312 36631733 10.1002/nop2.1312 PMC9834175

[16] De Albuquerque AMA, Costa LKDC, Mendonça AEOM, Souza MAS, Gouvéia BLAG, Torquato IMBT (2025) Escala ELPO de avaliação de risco para lesão em centro cirúrgico no intraoperatório: revisão integrativa. Revista Enfermagem Atual Derme 99(1):e025031. 10.31011/reaid-2025-v.99-n.1-art.2445

[17] Zeuschner P, Grosse Vollmer S, Linxweiler J, Wagenpfeil G, Wagenpfeil S, Saar M, Siemer S, Stöckle M, Heinzelbecker J (2021) Robot-assisted versus open radical nephroureterectomy for urothelial carcinoma of the upper urinary tract: A retrospective cohort study across ten years. Surg Oncol 38:101607. 10.1016/j.suronc.2021.101607 34022505 10.1016/j.suronc.2021.101607

[18] Yao Y, Wang H, Zhang Q, Teng H, Qi H, Zhang Q (2024) Learning curves for itinerant nurses to master the operation skill of Ti-robot-assisted spinal surgery equipment by CUSUM analysis: A pilot study. PLoS ONE 19(3):e0291147. 10.1371/journal.pone.0291147 38466746 10.1371/journal.pone.0291147 PMC10927145

[19] van der Schans EM, Hiep MAJ, Consten ECJ, Broeders IAMJ (2020) From Da Vinci Si to Da Vinci Xi: realistic times in draping and docking the robot. J Robotic Surg 14:835–839. 10.1007/S11701-020-01057-8 10.1007/s11701-020-01057-8 PMC7674320 32078114

[20] Andersson KK, Oksanen A, Falconer H, Brunes M (2025) From practice to perfection—complications and operative time learning curves in benign robotic-assisted laparoscopic hysterectomy. J Robot Surg 19(1):264. 10.1007/s11701-025-02429-8 40468095 10.1007/s11701-025-02429-8 PMC12137470

[21] Randell R, Greenhalgh J, Hindmarsh J, Alvarado N, Honey S, Kotze A, Pearman A, Dowding D (2021) How do team experience and relationships shape new divisions of labour in robot-assisted surgery? A realist investigation. Health 25(2):250–268. 10.1177/1363459319874115 31522572 10.1177/1363459319874115

[22] Wu J, Wang Y, Huang Y, Long X, Tang J, Gu D (2025) Learning curve analysis of extraperitoneal single-site robotic-assisted radical prostatectomy: a CUSUM-based approach. J Robot Surg 19(1):49. 10.1007/s11701-024-02202-3 39792294 10.1007/s11701-024-02202-3 PMC11723840

[23] Almeida FPT, Campos MEC, Castro PR et al (2022) Training in the protocol for robotic undocking for life emergency support (RULES) improves team communication, coordination and reduces the time required to decouple the robotic system from the patient. Int J Med Robot 18(6):e2454. 10.1002/rcs.2454 35998074 10.1002/rcs.2454

[24] Ballas D, Cesta M, Roulette GD, Rusnak M, Ahmed R (2018) Emergency Undocking in Robotic Surgery: A Simulation Curriculum. J Vis Exp 135:e57286. 10.3791/57286 10.3791/57286 PMC6101302 29863667

[25] Carlos G, Saulan M (2018) Robotic Emergencies: Are You Prepared for a Disaster? AORN J 108(5):493–501. 10.1002/aorn.12393 30376165 10.1002/aorn.12393

[26] Møller L, Olsen RG, Jørgensen L, Hertz P, Petersson J, Røder A, Konge L, Bjerrum F (2024) Training and education of operating room nurses in robot-assisted surgery: a systematic review. Surg Endosc 38(12):7024–7036. 10.1007/s00464-024-11335-3 39424704 10.1007/s00464-024-11335-3

[27] Møller L, Hertz P, Grande U, Aukdal J, Fredensborg B, Kristensen H et al (2023) Identifying curriculum content for operating room nurses involved in robotic-assisted surgery: a Delphi study. Surg Endosc 37(4):2729–2748. 10.1007/s00464-022-09751-4 36471061 10.1007/s00464-022-09751-4

[28] Nicolas Z, Eleonora L, Enrico M, Giovanni F (2025) More procedures, more efficiency: optimizing operating room during the phase of learning curve—experience of first 100 robotic bariatric procedures in a single center. J Robot Surg 19(1):233. 10.1007/s11701-025-02396-0 40411713 10.1007/s11701-025-02396-0 PMC12103475

